# An overview of the statistical methods reported by studies using the Canadian community health survey

**DOI:** 10.1186/1471-2288-14-15

**Published:** 2014-01-25

**Authors:** Dean W Yergens, Daniel J Dutton, Scott B Patten

**Affiliations:** 1 Department of Community Health Sciences, University of Calgary, Calgary, Alberta, Canada

**Keywords:** Health statistics, Statistical methods, Health survey, Population research, Canada, Canadian community health survey

## Abstract

**Background:**

The Canadian Community Health Survey (CCHS) is a cross-sectional survey that has collected information on health determinants, health status and the utilization of the health system in Canada since 2001. Several hundred articles have been written utilizing the CCHS dataset. Previous analyses of statistical methods utilized in the literature have focused on a particular journal or set of journals to understand the statistical literacy required for understanding the published research. In this study, we describe the statistical methods referenced in the published literature utilizing the CCHS dataset(s).

**Methods:**

A descriptive study was undertaken of references published in Medline, Embase, Web of Knowledge and Scopus associated with the CCHS. These references were imported into a Java application utilizing the searchable Apache Lucene text database and screened based upon pre-defined inclusion and exclusion criteria. Full-text PDF articles that met the inclusion criteria were then used for the identification of descriptive, elementary and regression statistical methods referenced in these articles. The identification of statistical methods occurred through an automated search of key words on the full-text articles utilizing the Java application.

**Results:**

We identified 4811 references from the 4 bibliographical databases for possible inclusion. After exclusions, 663 references were used for the analysis. Descriptive statistics such as means or proportions were presented in a majority of the articles (97.7%). Elementary-level statistics such as t-tests were less frequently referenced (29.7%) than descriptive statistics. Regression methods were frequently referenced in the articles: 79.8% of articles contained reference to regression in general with logistic regression appearing most frequently in 67.1% of the articles.

**Conclusions:**

Our study shows a diverse set of analysis methods being referenced in the CCHS literature, however, the literature heavily relies on only a subset of all possible statistical tools. This information can be used in identifying gaps in statistical methods that could be applied to future analysis of public health surveys, insight into training and educational programs, and also identifies the level of statistical literacy needed to understand the published literature.

## Background

The analysis of public health datasets such as the Canadian Community Health Survey (CCHS) is an active field of research. National-level surveys such as the CCHS, the United States National Health Interview Survey (NHIS), the United States Behaviour Risk Factor Surveillance System (BRFSS), and the Canadian General Social Survey (GSS) provide among the most important information about the health status on a national level on an on-going basis.

The CCHS is a national, cross-sectional survey conducted by Statistics Canada that collects information on health determinants, health status and the utilization of the health system in Canada, and represents approximately 98% of the population over age 12 [1]. The first cycle of the CCHS was conducted in 2001. Prior to 2008 the CCHS was conducted every two years, but since 2008 the survey has been conducted on an annual basis. CCHS datasets are released in a public use format through various academic and government institutions; Statistics Canada has included it as part of their Data Liberation Initiative, the goal of which is to make data available to researchers at post-secondary institutions [2]. Due to the depth (hundreds of variables are collected) and scope (often cycles have over 100,000 observations) of the CCHS, several hundred articles have been written utilizing different cycles of the CCHS dataset. However, the use and reporting of the statistical methods most associated with the analysis of the CCHS, and other large national surveys, has not been described in the health literature.

Several studies have looked at the prevalence of different statistical methods in specific journals, or groups of journals, generally with the goal of identifying the statistical knowledge needed for specific identifiable groups. Windish [3] evaluated medical residents’ understanding of statistical methods and the interpretation of research results through a questionnaire. That paper concluded that basic concepts of methods were not understood by most residents, yet complicated methods were being used in the literature. This knowledge gap was identified through a literature search of six general medical journals.

Becker [4] investigated the statistical methods and study designs used in original articles published in the South African Medical Journal. Becker concluded that biostatisticians and epidemiologists play a vital role when more complex statistical methods are used in the analysis. Scotch [5] examined statistical methods presented in the Journal of American Medical Informatics Association and the International Journal of Medical Informatics, concluding that biomedical informaticians should have a minimum understanding of descriptive and elementary statistics. Other reviews, Rao [6], Vanhanikkila [7] and Selvin [8], have additionally reported on the statistical methods described in other journals.

The literature is scarce on the statistical methods utilized by researchers using specific datasets, such as the CCHS, NHIS, BRFSS and other national surveys. Though these datasets are used by hundreds of researchers, there is little quantitative understanding of the statistical methods utilized and whether analytical techniques are changing over time. Additionally, given the cost of designing, implementing and managing these national public health surveys, it is essential to know how they are being utilized and analysed, since the results are used to influence public health policy decisions.

Our objective in this paper is to describe the statistical methods reported in the studies using the CCHS. Our search is not conditional on studies appearing in specific journals, only that the studies are indexed in commonly used bibliographical databases. We examine four core categories of analysis methods: descriptive statistics, elementary statistics, regression analysis methods, and machine learning algorithms. We also describe the use of statistical software identified in the articles and identify publication trends across time.

## Methods

### Literature search strategy

The first step of our study was to define reasonable boundaries for our search. The literature search was conducted for articles indexed in Ovid Embase, Ovid Medline, Web of Knowledge, and Scopus bibliographical databases. Our search strategy included the terms “Canadian Community Health Survey*” and “CCHS”. These terms were then combined with other keywords that could be associated with the survey including variations of the cycle (e.g. "Cycle 1.1", "Cycle 1.2", "Cycle 2.1" etc.), sub-cycle (e.g. "mental health and well-being", "Canadian Forces", "Healthy Aging", etc.) and the years of the survey (e.g. 2000, 2001, etc.) to identify potential references. References were restricted to articles published up to December 31, 2012. References were then retrieved and duplicates were removed. One reviewer (DWY) excluded any references that did not actually utilize the CCHS data. References were also excluded if they were missing the abstract, were a conference abstract or commentary, and in instances where an electronic readable Portable Document File (PDF) of the article was not available. Other than the exclusion of conference abstracts and commentaries, we did not put any restriction on types of publications we included such as papers classified as “original research” or “reviews”. Narrative reviews are increasingly being replaced by systematic reviews that often present statistical estimates and even statistical models (e.g. meta-regression). Given the goals of this project, reviews were considered eligible for this reason. The literature search was conducted on January 14, 2013.

A review of previous studies reviewing statistical methods [3-8] were collated and analysed by reviewers DWY and DJD. These papers followed different conventions when classifying their results. We opted for a combination of the classifications used by Windish [3] and Scotch [5]: methods could be classified as descriptive statistics, elementary statistics, regression techniques, and machine learning, each of which was associated with a series of “keywords”. Keywords for descriptive statistics included reporting a mean, standard deviation, median, interquantile range (IQR), counts (n/%), frequencies and/or cross tabulations. Keywords for elementary statistics included reporting a *t*-test, chi-square, Kaplan-Meier, Wilcoxon rank sum test, Fisher exact test, ANOVA, and ANCOVA. Keywords for regression techniques included linear, logistic, ordinal, non-linear, conditional, Poisson, binomial, and/or meta-regression. It is possible that the use of some statistical techniques were not detected by the automated search because they were not accurately described in the indexed manuscript. Following Scotch [5], we included a category of keywords indicating machine learning and data mining research, containing Bayesian networks, decision trees, artificial neural networks, and/or support vector machines.

### Data management and custom software

Once references were identified for inclusion by our literature search strategy, they were imported into a custom Java-based program that was used to facilitate the management of references and PDF documents. This software was created by DWY [9] and utilized for this project, hereafter “Synthesis” (http://www.synthesis.info). Synthesis uses the open-source Apache Lucene [10] database, which is a searchable database designed for the management and retrieval of textual information. Lucene has been applied to medical and biology Information Retrieval projects in the past [11,12]. Synthesis uses Lucene's text search abilities to find key words or phrases in an article, similar to what can be done in most commercially available word processors by using the "find" command. Lucene is also able to search information within tables, as long as the table has not been embedded in the PDF as an image. The software then summarized, counted, and organized the identified keywords. These organized results included bibliographic information for each document, the electronic copy of that document with keywords identified in the document, and identified keyword variables that can later be filtered or otherwise manipulated (similar to values stored in a spreadsheet program). Using this software greatly increased the speed with which this literature search and analysis could be completed, thereby increasing the volume of articles that could be feasibly searched.

The automated search for methods within the sampled literature was then further refined. Alternative keywords were included for many of the statistical methods after an initial analysis. This was a necessary refinement to address the issue of different phrasing to identify the same statistical method in text. For example, chi-squared tests can be identified in the text of a publication as “χ^2^”, “chi-square” or “chi-squared”. Additionally, proximity word searching was utilized where it was deemed appropriate. Proximity word identification enabled us to search for two words within a specified number of words with each other; in all cases it was limited to a range of 5 words. An example would be “linear regression” being counted if the software encountered the phrase “We used linear and logistic regressions…” but not “…linear trends were observed”.

To assess the automated coding approach, a random sample of articles was selected (n=56) from the included articles where one of the authors (DJD) manually coded the statistical methods from the PDFs. Agreement between the human reviewer and the computer was high. For example, there was 96% agreement regarding the presence of descriptive statistics in the sample (54/56). The agreement between the human reviewer and the computer algorithm had 100% agreement for the classification regression with logistic and linear regression having 94.6% and 92.9% agreement. Elementary statistics had an 83.9% agreement with the T-Test and Chi-squared tests agreeing 94.6% and 87.5% of the time. Descriptive statistics had a 94.4% agreement, with median reporting 82.1% and standard deviation at 80.4%. Mean reported the poorest level of agreement at 55.4%, most likely due to the various possible meanings of the word mean.

### Data analysis

All data analysis occurred in R Studio (version 0.96) [13] utilizing the R statistical language version 2.15 [14]. Only counts and frequencies of the methods used in the sampled literature were used in the analysis. All charts and tables were also produced in R Studio.

## Results

**Figure 1:**
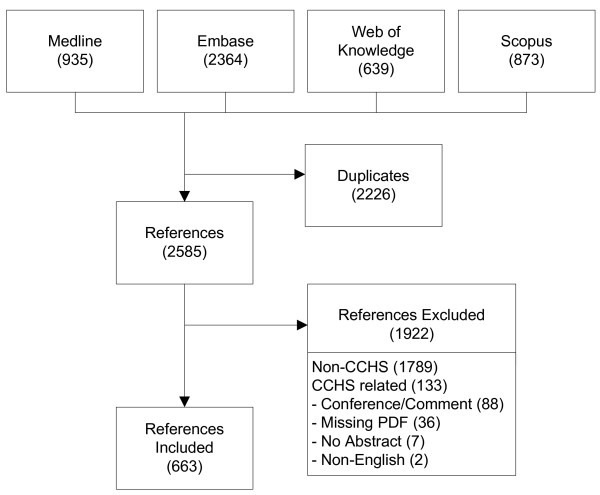
Flow diagram of the literature retrieval process (Number in brackets indicates number of references at each stage).

We identified 4811 references for inclusion. 2364 of the references came from Embase, 935 came from Medline, 639 came from Web of Knowledge, and 873 came from Scopus. After removal of duplicate references we were left with 2585 total references. We excluded 1789 references that did not actually use the CCHS data in their study. Another 133 references did use the CCHS data, but were excluded because they did not have an abstract (7), did not have a PDF associated with them (36), were conference abstracts or commentaries (88), or were not written in English (2). This left us with 663 references for analysis. Figure 1 shows the flow diagram of the literature retrieval process.

The statistical methods used in the articles are presented in Table 1. Descriptive statistics were present in the majority (97.7%) of statistical methods presented in the articles with count (n/%) data represented in 93.4% of the articles. Reporting the mean of a variable was popular (51.9% of papers), but a common distributional characteristic, the standard deviation, was less frequently reported (18.6%). Elementary statistics were less frequently used and referenced than descriptive statistics, identified in 29.7% of articles. The most frequent elementary statistical method was the chi-squared test (20.5%) followed by the *t*-test (10.0%). Regression analysis techniques were frequently mentioned in the articles, with 79.8% of articles using or referencing them. The most popular kind of regression was logistic regression (67.1%) followed by linear regression (12.4%). Multi-variate and multi-variable regressions accounted for 17.6% and 3.5% of the papers reviewed; these terms are likely to be applied in the same context: a regression with multiple regressors. In addition, several other regression keywords were represented in the literature including ordinal (1.1%), non-linear (0.8%), Poisson (2.6%), binomial (2.0%) and meta-regression (0.6%). The terms conditional (0.6%) and unconditional (0.3%) were also present in the literature reviewed. No machine learning keywords were identified during the analysis (not shown in Table 1).

**Table 1:** Statistical method breakdown

**Statistical method**	**Number (%)**
Descriptive statistics	648 (97.7%)
Mean	344 (51.9%)
Standard deviation	123 (18.6%)
Median	101 (15.2%)
Inter Quantile Range (IQR)	8 (1.2%)
Counts (n%)	619 (93.4%)
Frequencies	86 (13%)
Cross tabulations	89 (13.4%)
Elementary statistics	197 (29.7%)
T-Test	66 (10%)
Chi-Square	136 (20.5%)
Correlation (Pearson/Rank)	16 (2.4%)
Kaplan-Meier	2 (0.3%)
Wilcoxon	0 (0%)
Fisher exact test	4 (0.6%)
ANOVA	14 (2.1%)
ANCOVA	13 (2%)
Regression	529 (79.8%)
Multi-variable	23 (3.5%)
Multi-variate	117 (17.6%)
Logistic	445 (67.1%)
Linear	82 (12.4%)
Ordinal	7 (1.1%)
Non-Linear	5 (0.8%)
Conditional	4 (0.6%)
Unconditional	2 (0.3%)
Poisson	17 (2.6%)
Binomial	13 (2%)
Meta	4 (0.6%)

We also organized studies by their choice of statistical software. A breakdown of the statistical program information is presented in Table 2. The most popular software referenced was SAS (30.8%), with STATA (13.1%) in second place and SPSS (12.8%) in a close third. Other statistical programs mentioned were SUDAAN (6.5%), MLWin (2.0%) and WestVar (0.6%). It was a common convention in these articles to mention several statistical programs with the most frequent combination being SAS and SUDAAN. Upon further analysis (not presented) we found that the majority references of the SUDAAN statistical application was the result of being used in conjunction with SAS for its callable bootstrapping procedures [15,16].

**Table 2:** Statistical software breakdown

**Software application**	**Number (%)**
SAS	204 (30.8%)
SPSS	85 (12.8%)
STATA	87 (13.1%)
SUDAAN	43 (6.5%)
MLWin	13 (2%)
West Var	4 (0.6%)

**Figure 2:**
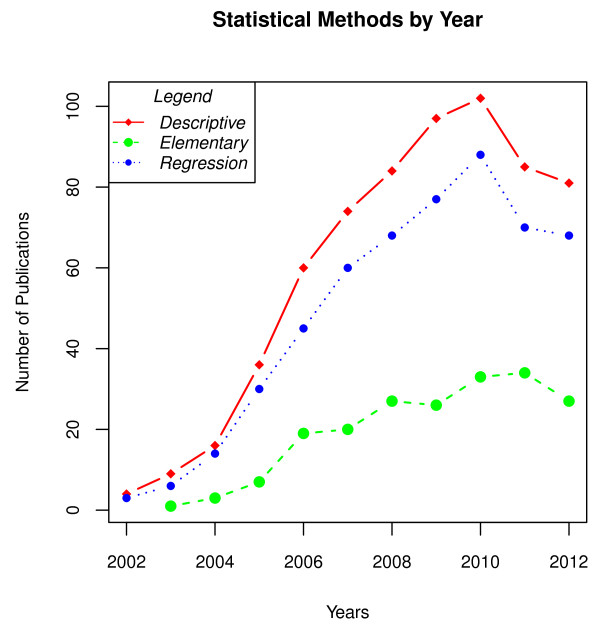
Statistical methods by year of publication, grouped into general categories of ‘Descriptive’, ‘Elementary’ and ‘Regression’.

Figure 2 presents the three categories of descriptive, elementary, and regression across the publication date years 2002 to 2012. Figure 2 shows that reporting descriptive and regression statistical methods increased at a faster rate than elementary statistics, and the distance between elementary statistics and the rest grew over time.

## Discussion

This study described which statistical methods have been referenced in the literature using the Canadian Community Health Survey (CCHS) dataset. Descriptive statistics and regression analysis methods dominate the literature in comparison to elementary statistics. The high prevalence of descriptive statistics is unsurprising, since papers using the CCHS are almost all presenting some analysis or description of the data, and descriptive statistics are usually the first table in quantitative papers.

However, the magnitude of the difference in prevalence of reported elementary statistics versus regression techniques is striking.

There are two possible reasons for the large difference in prevalence between elementary statistics and regression techniques: modelling has become the only step of analysis in many empirical papers, or, authors do not specifically mention elementary statistics when employing them. The first point, that models are the main analysis step taken by most researchers, is one that is controversial to some practitioners. Nevertheless, the epidemiological paradigm is to analyze exposures and outcomes while considering effect measure modification and confounding, thus modelling is a natural tool to employ [13]. In the social sciences, consideration of statistical effects necessitates controlling for covariates to curtail omitted variables bias, which is mathematically the same issue as confounding. Thus, a series of elementary statistics might not be necessary, or might be redundant, when the research question of interest naturally lends itself to modelling. The second point, that elementary statistics are not explicitly reported, likely explains a large share of the gap between regression techniques and elementary statistics. The elementary statistics are likely not reported themselves, but used in regression interpretation and coefficient hypothesis testing. For instance, any linear regression that reports a statistically significant coefficient is actually reporting a t-test of that coefficient being equal to zero, the authors just do not identify it as such, probably because most readers understand the hypothesis test being identified. This is important, since an individual would need to understand elementary statistical tools to be able to engage with a study using regression techniques, even though that paper does not specifically mention those elementary statistics.

Of the regression techniques utilized, this study shows that logistic regression is the most common technique employed by studies using the CCHS. In most health-condition-outcome models, an individual either has a health condition or they do not, and in that case a logistic regression is a popular choice due to its mathematical properties, such as no upper bound existing for the log of an odds. There is also a tendency to dichotomize continuous variables so that they may serve as an outcome variable in a logistic regression model, either because of medical ease of interpretation of the variable (e.g., dichotomizing body mass index into “obese” or “not obese”) or because researchers are more comfortable with logistic regressions (working with them or presenting them to an audience familiar with them). It is interesting that one regression technique, logistic regression, should be present in nearly 70% of the papers included in our literature search, and is indicative of the importance of understanding this regression technique for anyone interested in health research.

Figure 2 shows time trends of the three broad types of methods observed in the data. One notable point is that the number of papers using the CCHS increased from 5 in 2002 to over 100 in 2010, which represents an impressive feat of data dissemination by Canadian academic institutions and Statistics Canada. The statistical results used by year kept a steady pattern, with descriptive statistics being present in most papers, regression techniques closely trending alongside descriptive statistics, and elementary statistics trending much lower. The year 2010 looks like it was a peak in research using the CCHS surveys, and the number of publications starts to plateau after 2010 with the three methodological categories maintaining their relative ranks.

The statistical software preferences illustrated the popularity of SAS in the analysis of the CCHS datasets. The prevalence of SAS is unexplained by this paper; however, it might have to do with SAS being a prominent application in government environments or being available in the Statistics Canada Research Data Centres (RDC) where access to the CCHS micro data is available. Additionally, SAS provides macros for tasks such as bootstrap variance estimation which is often applied in the analysis of the CCHS. Even though this paper focused on the use of statistical methods and the associated statistical software, other software was identified during our review, such as in the analysis of geographical information (i.e. ESRI ArcGIS) [17], dietary analysis (i.e. SIDE-IML, SIDE, C-SIDE) [18] and discrete event simulation (i.e. Arena) [19].

Our study offers a new approach to the description of statistical methods presented in the literature. While the previous reviews of statistical methods used in health literature have focused on the analysis of articles indexed in specific journals or sets of journals, we investigated how a specific dataset was utilized in the reporting of statistical methods. Articles were drawn from 233 journals and spanned many specialties. This study describes the statistical techniques that a reader must be familiar with in order to understand the information within articles using a popular population-level dataset. With the growing popularity of open access journals, statistical literacy of the general public might be an issue that needs to be addressed in knowledge translation. When regression models are the norm for presenting results by researchers, the message within the papers might not be nuanced effectively or clearly understandable to media or interested individuals.

Even though this study only focused on the statistical methods reported in the literature and not the actual statistics used in the analysis, this information may be useful in identifying research gaps. For example, we could not identify any machine learning methods such as Artificial Neural Networks, Support Vector Machines or Random Forests in the analysis of CCHS data, even though these machine learning algorithms are used in the analysis of other medical areas such as mortality prediction and health services utilization.

There are several limitations to our study. First, we only reviewed references from the Ovid databases Medline and Embase, Web of Knowledge, and Scopus bibliographic databases. The CCHS dataset could have been applied to other disciplines, such as computer science, not fully indexed in the selected bibliographic databases and that literature would not be reflected in our analysis. Additionally, our study only looked at the bibliographical databases and would not include grey literature such as government reports. Second, in our retrieval from the bibliographical databases, if the CCHS were identified as used in the article, but not the abstract, it would not be included in our analysis. This is common for all literature searches that use the abstract as the basis of the search, such as a search in PubMed. In terms of the analysis, the search function only finds textual information, so if a statistical method was used in a table formatted as a picture, or referenced solely in a footnote that was a picture, the statistical method may not have been found by the software. We felt this was unlikely, as most methods sections of articles tend to report the statistical techniques and datasets used. Another limitation was in the development of the terms and phrases used for the automated identification of statistical concepts as there are many ways to phrase things in the English language and some statistical terms can be used in other contexts. For example, the word “mean” was identified as an arithmetic average, but the word “mean” can appear in the text and not be referring to an average, which the software would not be able to discriminate. Further complicating this issue, there is no standardized reporting tradition in health statistics. For instance, awkward turns of phrase to describe a mean, while obviously referring to a mean, were missed by the algorithm. An example would be a phrase like “respondents were averaged 20 years of age”, which is reporting a descriptive statistic but is not captured by the search process.

## Conclusions

This study describes which statistical methods have been referenced in a large population-level health dataset. As public health data is released to researchers, the ability to convey which statistical methods have been applied to these datasets provides valuable information for epidemiologists and biostatisticians. Even though the emphasis here has been placed on the statistical literacy of the readers, the results are also of interest to the researchers and developers of custom analysis software and those who run instructional courses that may be needed to educate the statistical methods being applied. Given the cost of implementing and managing large national public health surveys, it is important to understand how these datasets are being utilized and analysed.

## Competing interests

DWY is a co-founder of Synthesis Research Inc which owns the intellectual property for the Synthesis software application. DJD and SP declare they have no competing interests.

## Authors’ contributions

DWY contributed to the study design, software development, analysis and the writing of the manuscript. DJD contributed to the study design, analysis, and editing of the manuscript. SP contributed to the study design and was integral in all aspects of the project and the writing of the manuscript. All authors reviewed and provided comments on the final manuscript. All authors read and approved the final manuscript.

## Pre-publication history

The pre-publication history for this paper can be accessed here:

http://www.biomedcentral.com/1471-2288/14/15/prepub
